# Atypical Audiovisual Speech Integration in Infants at Risk for Autism

**DOI:** 10.1371/journal.pone.0036428

**Published:** 2012-05-15

**Authors:** Jeanne A. Guiraud, Przemyslaw Tomalski, Elena Kushnerenko, Helena Ribeiro, Kim Davies, Tony Charman, Mayada Elsabbagh, Mark H. Johnson, the BASIS Team

**Affiliations:** 1 Centre for Brain and Cognitive Development, Department of Psychological Science, Birkbeck, University of London, London, United Kingdom; 2 Institute for Research in Child Development, School of Psychology, University of East London, London, United Kingdom; 3 Centre for Research in Autism and Education, Department of Psychology and Human Development, Institute of Education, University of London, London, United Kingdom; The University of Western Australia, Australia

## Abstract

The language difficulties often seen in individuals with autism might stem from an inability to integrate audiovisual information, a skill important for language development. We investigated whether 9-month-old siblings of older children with autism, who are at an increased risk of developing autism, are able to integrate audiovisual speech cues. We used an eye-tracker to record where infants looked when shown a screen displaying two faces of the same model, where one face is articulating/ba/and the other/ga/, with one face congruent with the syllable sound being presented simultaneously, the other face incongruent. This method was successful in showing that infants at low risk can integrate audiovisual speech: they looked for the same amount of time at the mouths in both the fusible visual/ga/− audio/ba/and the congruent visual/ba/− audio/ba/displays, indicating that the auditory and visual streams fuse into a McGurk-type of syllabic percept in the incongruent condition. It also showed that low-risk infants could perceive a mismatch between auditory and visual cues: they looked longer at the mouth in the mismatched, non-fusible visual/ba/− audio/ga/display compared with the congruent visual/ga/− audio/ga/display, demonstrating that they perceive an uncommon, and therefore interesting, speech-like percept when looking at the incongruent mouth (repeated ANOVA: displays x fusion/mismatch conditions interaction: *F*(1,16) = 17.153, *p* = 0.001). The looking behaviour of high-risk infants did not differ according to the type of display, suggesting difficulties in matching auditory and visual information (repeated ANOVA, displays x conditions interaction: *F*(1,25) = 0.09, *p* = 0.767), in contrast to low-risk infants (repeated ANOVA: displays x conditions x low/high-risk groups interaction: *F*(1,41) = 4.466, *p* = 0.041). In some cases this reduced ability might lead to the poor communication skills characteristic of autism.

## Introduction

Autism is a neurodevelopmental disorder typically diagnosed from around 3 years of age, which is characterized by impaired communication and social skills and repetitive or stereotypical behaviours [1]. An estimated 10% of children with autism never develop functional language skills [2], showing deficits in both understanding and producing language [3]–[4]. Communication impairments in individuals with autism can range from severe language delay to relatively intact language accompanied by problems with functional communication [5].

It is well established that autism is highly heritable [6], but little is known about the underlying process through which symptoms emerge (for a review see [7]). Specifically, the developmental processes that underlie the emergence of the poor language abilities characteristic of autism are unknown. Recently, an electrophysiological study showed that the influence of visual speech cues on the auditory processing of language is reduced in adolescents with autism, and that the strength of this influence correlates with their social communication skills [8]. Individuals with autism may not be able to make use of the crossmodal, audiovisual cues that facilitate speech perception (as shown in neurotypical adults [9] and in typically developing children [10]), and which are considered to be crucial in native language acquisition [11] and thus facilitate development of communication skills in general. Similarly to blind children whose inability to integrate audiovisual information is thought to affect their language development [12], it is possible that impairment in this basic skill in infants at high risk for autism leads to language delays. Infants who are genetic relatives of children with autism might share some characteristics with affected individuals; even if around 80% do not themselves go on to receive a diagnosis [13]. In adults, the Broader Autism Phenotype (BAP) refers to clinical, behavioural and brain characteristics associated with autism found not only in affected individuals, but also in their relatives [14]. It is not known whether reduced ability to integrate audiovisual (AV) information is a feature of an early form of the BAP, and/or whether it is involved in the emergence of language difficulties in children with autism.

Several behavioural studies have been conducted to investigate whether integration of AV speech information is reduced in autism. Adolescents with autism display weaker lip-reading skills and are less able to integrate matched AV speech in the context of auditory noise when compared with typical controls [15]. Integration of audiovisual speech information in children with autism has often been investigated with a McGurk paradigm, where differing auditory and visual inputs are presented [16]. While children with autism often show deficits in crossmodal integration (for a review see [17]), studies using the McGurk paradigm have reported conflicting results: several studies show that children with autism are less influenced by visual speech than those with typical development [18]–[20], even when time spent looking at the face of the speaker was controlled [21], while others have suggested that children with autism demonstrate normal AV integration of speech stimuli [22], when they are able to lip-read [23]. Nevertheless, an inverse association exists between AV speech processing abilities and social impairment in children with autism [24], suggesting that an impaired ability to integrate AV speech information might play a role in social difficulties faced by these children, possibly because of difficulties in their language and communication development resulting from impaired AV speech integration skills.

In the present study, we investigated whether 9 month-old infants at high-risk of developing autism have difficulties integrating AV speech information. We used the same rationale as in the Kushnerenko et al. study (2008) [25], in which they showed that 5 month-old infants growing up in native English speaking families can integrate AV speech cues, and detect incongruent and non-fusible AV speech cues in the McGurk paradigm. In this study, infants’ neural responses to congruent visual and auditory information (visual/ba/− audio/ba/, and visual/ga/− audio/ga/) were compared with neural responses to two incongruent stimuli types: (1) A fusion condition, in which a face articulating the syllable/ga/is presented with incongruent auditory information/ba/; this is known to generate an English syllable-like fused percept “da” or “tha” in both children and adults; and (2) a mismatch condition (visual/ba/− audio/ga/), which is known to generate a non English syllable-like mismatched percept “bga” in both children and adults [16]. Infants’ neural activity in the fusion condition was similar to that generated by congruent displays, suggesting that they were integrating incongruent AV cues and perceiving a syllable. However, they showed different responses in the mismatched condition, suggesting that they were detecting the incongruence between cues from each modality. This paradigm was further adapted by Kushnerenko and Tomalski for use with an eye-tracker [26], [27]. In the present study we used preferential looking times to the mouths of congruent vs. incongruent stimuli, as attention to the mouth during articulation may be necessary in order to perceive a McGurk effect [28]. While orienting to the mouth may not be critical for this effect in adults [29], the reduced sensitivity of infants outside their foveal visual field may make fixation of the mouth critical [30]. Low risk infants demonstrated that they can integrate AV speech information, as they looked as long at the mouth in the fusion condition as in the congruent condition, and perceive incongruence in AV speech information, as they looked longer at the mouth in the mismatch condition than in the congruent condition. In contrast, the group of high-risk infants had the same looking behaviours in both the mismatch and fusion conditions, reflecting an absence or weakened AV integration and reduced ability to detect incongruence in AV cues.

## Materials and Methods

### Ethics Statement

The study was approved by London NHS (National Health Service) Research Ethics Committee (reference number: 06/MRE02/73) and conducted in accordance with the Declaration of Helsinki (1964). Parents gave their written informed consent for their infant to participate in the study.

### Participants

High and low-risk infants were recruited and tested across the same time window. We tested 31 high-risk infants (13 females) and 18 low-risk infants (10 females) both from the British Autism Study of Infant Siblings (BASIS; www.basisnetwork.org). Sample sizes were determined beforehand on the basis of power analyses from previous studies and our own pilot data.

The high-risk infants had an older full sibling (‘proband’ of which 4 were females) with a community clinical diagnosis of autism or autism spectrum disorder. Proband diagnosis was confirmed by an expert clinician (TC) based on information using the Development and Wellbeing Assessment (DAWBA) [31] and/or the parent-report Social Communication Questionnaire (SCQ) [32]. The low-risk infants had at least one older full sibling and no reported family history (1st degree relative) of autism.

The infants live in an English-language environment only. Groups were matched for ethnicity as much as possible: Most of the infants are white British, a couple infants are also white but not British (1 high-risk infant, 1 low-risk infant), and some have African (3 high-risk infants, 1 low-risk infant), or both white and Asian (1 low-risk infant) origins.

Infants were tested at around 9 months and 10 days of age (±26 days) in both groups. In another study we showed that 9 month-old infants at high-risk for autism do not have impaired auditory processing as the amplitude of their neural responses to white noise does not differ to the one of low risk infants’ [33]. Moreover, none of the parents reported that their child has a known or diagnosed hearing loss at 14 months old.

### Procedure

Infants sat on their parent’s lap in front of a TobiiT120 eye-tracker monitor (17′), at a distance of approximately 60 cm. Eye movements were monitored during recording through Tobii Studio LiveViewer. Calibration was carried out using 5 points: in the centre, top and bottom corners of the screen. Before presentation of each block the infants’ attention was focused on the centre of the screen using a colourful animation accompanied by a sound, which terminated once the infant fixated it.

### Preferential Looking McGurk Task

The same conditions and stimuli as in [25] were used: a mismatch condition with visual/ba/and auditory/ga/, which integrate to produce a non-English percept “bga”, and a fusion condition with visual/ga/and auditory/ba/, which integrate to produce an English syllabic percept “da” or “tha” [16]. Video recordings of a female native English speaker’s face articulating/ba/and/ga/sounds were edited to create incongruent instances of speech sound articulation by mixing the audio track with the incongruent articulation. The incongruent AV stimuli were presented to five native adult English speakers to test whether they produce illusory percepts [25]. Four of them reported hearing/da/or/ta/for VgaAba (fusion percept) and either/bga/or mismatched audiovisual input for VbaAga, and one adult reported only the auditory component in both situations. The presentation of these stimuli was adapted for use with the eye-tracker. We presented the stimuli in a preferential looking task with an incongruent face (mouthing/ba/in the mismatch condition, and/ga/in the fusion condition) being displayed on one side of the screen, along with the corresponding congruent face (mouthing/ga/in the mismatch condition, and/ba/in the fusion condition) on the other side of the screen. As directing visual spatial attention towards a face in a McGurk preferential display increases the influence of that face on auditory perception [34], we expected the infants to perceive a McGurk effect when looking at the incongruent face, and to hear the syllable being presented auditorily to them when looking at the congruent face. The position of the faces was pseudo-randomized across infants so that when the incongruent face was on the left side of the screen in the mismatch condition, it would be on the right side in the fusion condition (and vice-versa). Two blocks of 15 repetitions each were presented, one block showing the mismatch condition (congruent face next to mismatch face) and the other block showing the fusion condition (congruent face next to fusion face). The order of presentation of the blocks was counterbalanced across infants so that the same number of low- and high-risk infants saw the mismatch condition first and second, and the mismatch face on the left and right sides of the screen. Articulation of each face in a display was synchronized to the speech sound onset on every repetition by adjusting the sound at 360 ms from the stimulus onset. The auditory syllable lasted for the following 280–320 ms. Each single clip lasted 760 ms, and each block was 12 s long. The video stimuli were rendered with a digitization rate of 25 frames per second. Stereo soundtracks were digitized at 44.1 kHz with 16-bit resolution. For more information on stimuli see [25].

**Figure 1:**
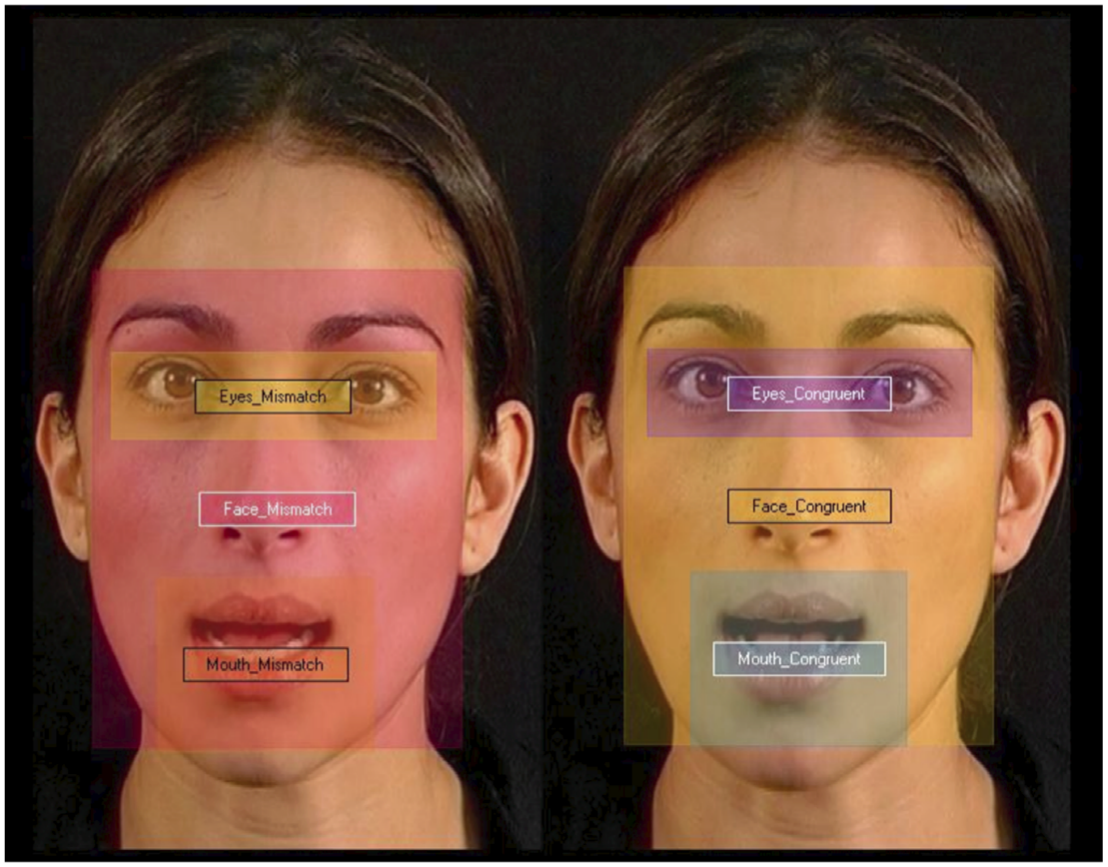
Stimuli and Areas of Interest (AOIs) in a mismatch display. The face on the left side is incongruent with the sound (/ga/) and mouths/ba/, which is known to create a non-fused percept ‘bga’ in children and adults. The face on the right side is congruent with the sound (visual/ga/- audio/ga/).

### Analysis

The eye-tracker data were analysed according to specific Areas-Of-Interest (AOIs) around the mouth, eyes, and face. Figure 1 illustrates the stimuli and AOIs chosen for our analysis. The AOIs were defined before collecting the data and independently to the ones chosen by Kushnerenko and Tomalski for the studies being conducted simultaneously in their own laboratory [26]–[27]. The total fixation length was calculated off-line for each infant and each AOI using the Tobii Studio software package and Tobii fixation filter (Tobii Inc.). As the time spent on each AOI varies within infants, we compared the time spent on mouths as a percentage of total time spent on the parts that are the most looked at in a speaking face, i.e. mouth and eyes of each face within each display. We investigated whether face scanning in general differed across groups using two-tailed independent sample t-tests for time spent on mouths, eyes, and faces. A two-way repeated ANOVA was used in the low-risk infant group to investigate whether time spent looking at the mouth in the congruent face was different to time looking at the mouth in the incongruent face and whether this effect depends on the type of incongruency, i.e. whether the AV speech cues are fusible (fusion condition) or not (mismatch condition). This analysis enabled to show that, while low-risk infants look longer at the mouth in the mismatched display than the mouth in the congruent display, showing that they can detect the incongruence between cues from each modality, they look as long at the mouth in the fusion display as at the mouth in the congruent display, suggesting that they perceive a syllable in both cases. Once we could show evidence that low-risk infants are sensitive to AV speech information correspondence using the preferential eye-tracking technique, we conducted another two-way repeated ANOVA in the high-risk infant group to look at whether the same effect could be found with this group. The differences between the groups were further investigated by adding group (low- vs. high-risk infants) as a between-subject factor to the repeated ANOVA.

## Results

Infants were excluded from the analysis if they only looked at one of the faces for the entire duration of the trial, i.e. one low-risk infant and five high-risk infants. All the other infants looked at both faces for at least 10% of the entire duration of the trial. Infants at low-risk looked at faces for about 10.9 seconds (±1.1 s) in the mismatch condition, and 10.1 seconds (±2.3 s) in the fusion condition. Infants at high-risk looked at faces for about 9.6 seconds (±2.7 s) in the mismatch condition, and 9.3 seconds (±2.7 s) in the fusion condition.

### No Difference in Face Scanning between High-risk and Low-risk Infant Groups

Children with autism have been reported as looking at faces in atypical ways [21], looking less at faces [35], eyes [36], and eyes and mouths [37] than typically developing children. Other studies have found that autistic children look more at mouths than typically developing children [38]. Differences in scanning faces have also been found in the unaffected adult siblings of individuals with autism [39]. It was critical to ascertain that no such differences would be present in the current sample, as this could affect the interpretation of our results. We found no significant group difference in time spent looking towards faces, eyes, and mouths (two-tailed independent sample t-test: t(41) = 1.853, *p* = 0.071 for faces; t(41) = 0.851, *p* = 0.4 for eyes; t(27) = 0.744, *p* = 0.463 for mouths). Average looking times to AOIs within both groups are summarized in Table 1.

**Table 1:** Average looking times to faces, eyes, and mouths across displays in infants at low- and high-risk.

Groups	Faces	Eyes	Mouths
Low-risk infants	10.5 s (±1.4 s)	1.4 s (±2.7 s)	7.2 s (±3.5 s)
High-risk infants	9.5 s (±2.4 s)	0.9 s (±1 s)	6.5 s (±2.5 s)

### Low-risk Infants can Integrate AV Speech Cues

**Figure 2:**
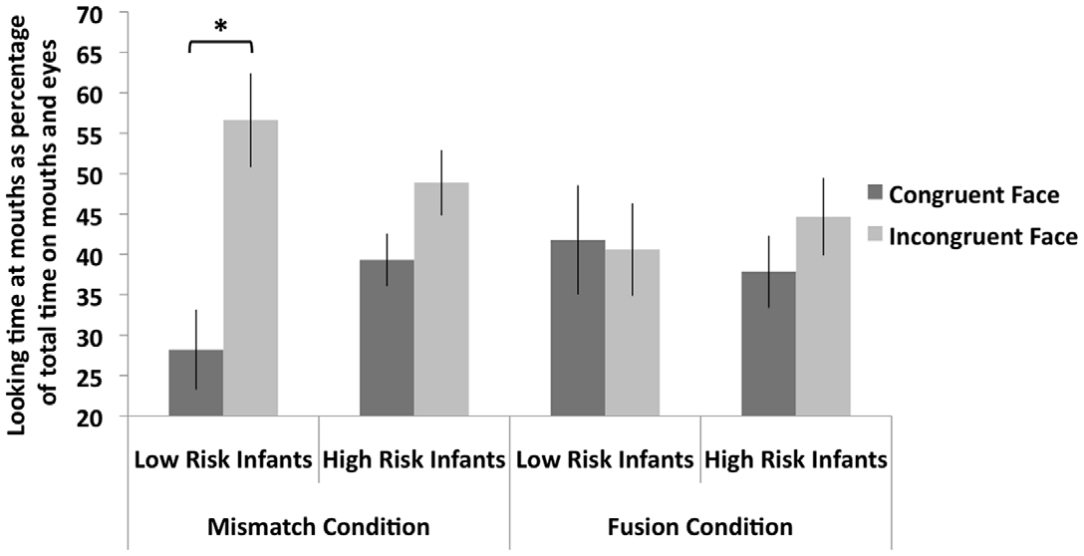
Looking time of infants at low versus high risk for autism in a McGurk paradigm. Low-risk infants looked as long at the incongruent mouth as at the congruent mouth in the fusion condition, demonstrating that they can integrate AV speech information, and they looked longer at the incongruent mouth than at the congruent mouth in the mismatch condition, indicating that they perceive incongruent, non-fusible AV speech information. In contrast, high-risk infants had the same looking behaviours in both the mismatch and fusion conditions, reflecting poor AV integration and detection of incongruence between AV information. Error bars are standard error of the means. *p<0.05.

As illustrated in Figure 2, we found that infants at low risk of developing autism looked longer at the mouth of the incongruent face than the congruent face in the mismatch condition, reflecting interest in an incongruent audiovisual combination, and looked equally long at the mouths of the incongruent and congruent faces in the fusion condition, showing that they perceived commonly heard English syllables when watching both faces in this condition (2-way repeated ANOVA, face type x condition interaction: *F*(1,16) = 17.153, *p* = 0.001). These data are in line with [25] and suggest that infants at low risk do perceive the fusion condition as an integrated percept similarly to adults, whereas an audiovisually mismatched percept is probably processed as a novel display.

### Reduced Ability to Integrate AV Speech Cues in High-risk Infants

Infants at risk of developing autism did not look significantly longer at the mouth of the incongruent face in either the fusion, or the mismatch condition (2-way repeated ANOVA, face type x condition interaction: *F*(1,25) = 0.09, *p* = 0.767). Further analysis confirmed that high-risk infants looked for equally long at the mouth in each condition in contrast to low-risk infants who looked longer at the mouth of the incongruent face in the mismatch condition only (repeated ANOVA, face type x condition x group interaction: *F*(1,41) = 4.466, *p* = 0.041).

## Discussion

Our data show that 9 month-old infants at low risk for developing autism looked longer at the mouth in the mismatch condition (visual/ba/− audio/ga/), than at the mouth in the congruent condition (visual/ga/− audio/ga/). This finding indicates that they can detect the incongruence between visual and auditory speech cues and orient their attention to ‘atypical’ audiovisual combinations. On the contrary, the infants at high risk for developing autism looked at the congruent and incongruent articulations in the mismatch display for equal amount of time. Our results suggest that high-risk infants have either a reduced ability to match audiovisual speech information or lack of interest in unusual communication patterns.

Our study enabled us to investigate whether the proposition based on the perceptual learning theory [40] that lack of attention to a speaker’s face deprives a child with autism of the experience necessary to develop typical sensitivity to visual speech information could also help understand atypicalities in integrating AV speech information in infants at high-risk. Lack of experience of looking at speaking faces can result in a weaker McGurk effect: Japanese individuals, who have been raised in a culture where looking at the face of the person one is speaking with is generally avoided, have been found to demonstrate weaker McGurk effects than American individuals [41]–[42], and have no change in the strength of their McGurk effect as they age [43] contrary to English children (e.g., [16] [44]). Noteworthy, 19% of the infants at high-risk were excluded from analysis because they did not look at one of the faces in the preferential display, against 6% only in the group at low-risk. Similarly to children with autism infants at high-risk may show reduced social gaze to others’ faces when speech is produced [35], and might pay less attention to the face in general [45]. Infants at high-risk tended to look less at speaking faces than infants at low-risk in our study (*p* = 0.071). Our study therefore suggests that lack of attention to speaking faces plays a role in preventing AV integrative abilities to develop in infants at risk in the first place.

The various reasons proposed to explain reduced AV integration in children with autism could easily be offered to further explain the difficulties we found in at-risk infants. Infants at risk and children with autism might have common BAP characteristics such as deficits in attending to multimodal information [18]. Children with autism have structural abnormalities in the cerebellums causing disruption in attentional systems, which if infants at high-risk also have might particularly impair their ability to shift attention within the visual modality and between auditory and visual modalities as in individuals with autism (e.g., [46]). Children with autism are known to have broader executive function deficits [47], which if shared by infants at high-risk would prevent them from coordinating different sources of information from different modalities. Children with autism and infants at high-risk might also have in common abnormal processing of unimodal social information, such as atypical processing of vocal sounds by superior temporal sulcus voice-selective regions [48]. Such impairment might in turn affect the integration of information from another modality, learning of language, and/or alter the perception of the auditory stimuli preventing infants at high-risk from doing the task. It will be important to control for these various factors in future similar work in siblings of children with autism by for instance investigating whether infants at high- and low-risk have similar reaction times when switching from looking at a toy moving in front of them to a sound played to them from another location and similar characteristics of evoked potentials (amplitude, latency, and topography) when presented with vocal stimuli only or in combination with visual cues.

Studies using the McGurk paradigm have shown that AV speech perception plays an important role in speech production. AV speech perception is related to spontaneous babbling in infants, and speech production in preschoolers [49]–[50], possibly because visual information about speech articulation not only enhances phoneme discrimination, but also contributes to the learning of phoneme boundaries in infancy [51]. Given the potentially important role of both visual and auditory speech perception in language development, a deficit in AV speech processing may contribute to the significant language delays often found in children with autism [5] and in infants who will go on to receive a diagnosis or show features of the BAP. Autistic-like characteristics, such as the inability to detect inter-modal correspondence of facial and vocal affect [52] could also possibly result from a deficit in integrating AV information. Impaired integration of AV information might thus play a crucial role in the language and social difficulties of individuals with autism.
